# Frequency and characteristics of promissory conference abstracts, i.e. abstracts without results, accepted at Cochrane Colloquia 1994-2020

**DOI:** 10.1186/s12874-021-01442-3

**Published:** 2021-11-08

**Authors:** Darko Novak, Livia Puljak

**Affiliations:** Center for Evidence-Based Medicine and Health Care, Catholic University of Croatia, Ilica 242, 10000 Zagreb, Croatia

**Keywords:** Promissory abstracts, Research conference, Knowledge dissemination, Cochrane

## Abstract

**Background:**

The purpose of a conference abstract is to summarize the main points of a research-related report that will be presented at an academic conference. However, some conferences accept and publish abstracts without results, which is contrary to the basic idea of a conference abstract as a dissemination tool. A conference abstract without results included is called a “promissory abstract”. This study aimed to analyze the frequency and characteristics of promissory conference abstracts, i.e. abstracts submitted without results, accepted at Cochrane Colloquia.

**Methods:**

We analyzed 8297 conference abstracts accepted at 25 Cochrane Colloquia, organized in 1994–2020, which were publicly available on the website of the Cochrane Library. Two authors screened abstracts to identify promissory abstracts. We extracted characteristics of promissory abstracts.

**Results:**

Among abstracts accepted for Cochrane Colloquia, 8.7% were promissory; 475 (66%) were accepted as poster presentations, 241 (34%) as oral presentations and 1 as a workshop. The median number of authors in promissory abstracts was 4 (interquartile range: 3 to 6 authors). In 245 (34%) promissory abstracts, affiliations of authors were not reported. The authors were most commonly affiliated with the following countries: UK (472; 36%), Canada (*N* = 123; 26%), China (*N* = 76; 16%), United States (*N* = 66; 14%) and Australia (*N* = 53; 11%). There were 512 (71%) promissory abstracts in which study design was not reported.

**Conclusion:**

Promissory abstracts were commonly accepted at Cochrane Colloquia. Such abstracts deserve further attention, as they are detrimental in terms of the dissemination of new knowledge presented at a conference. Conference organizers could ask authors to update the abstract results subsequently to enable the dissemination of information presented at a conference.

**Supplementary Information:**

The online version contains supplementary material available at 10.1186/s12874-021-01442-3.

## Background

Research conferences are attended by individuals sharing a common interest, wishing to learn something new from their peers and to share their results. Sharing of knowledge and ideas is usually the main purpose of research conferences [1].

Conference abstracts are important tools in sharing knowledge and ideas, as they are usually written long before the conference date and submitted in hopes that scientific committees will choose them for a conference presentation. Conference guidelines determine the format of a conference abstract, but generally, researchers are expected to provide a brief background to their research topic, study aim, methods, main results and conclusions. Depending on the conference, it may be acceptable to submit a conference abstract for preliminary results or even for work that is not finished, but it is expected to be completed by the time the conference takes place [1].

A conference abstract without results included is called a “promissory abstract”. This “promissory abstract” concept can be found in instructions for authors of conferences that accept promissory abstracts (an example: [2]). Additionally, it is mentioned occasionally in instructions on writing research abstracts [3].

A Merriam-Webster dictionary defines the word “promissory” as “*containing or conveying a promise or assurance*” [4]. A significant problem with promissory abstracts is that such abstracts will be included in an abstract book, and then audiences that did not have a chance to attend that specific conference oral or poster presentation will be deprived of the main study results presenter will show at the conference. This has important implications for disseminating research findings, as conference abstracts may be relevant first sources of new research information. Thus, the promissory abstracts may be contrary to the idea of conference abstracts being used as a scientific dissemination tool.

Cochrane is an international network of individuals committed to producing and disseminating high-quality systematic reviews regarding health care. Cochrane also plays a key global role in developing new methods in evidence synthesis [5]. The Cochrane methodology is considered the gold standard and is frequently used as a model in the research field of evidence synthesis and research methodology. Thus, it is important to assess research practices within Cochrane.

This study aimed to analyze the frequency and characteristics of promissory conference abstracts, i.e. abstracts submitted without results, accepted at 25 Cochrane Colloquia in the period 1994–2020.

## Methods

### Study design

This was a cross-sectional analysis of a sample of conference abstracts.

### Sample

We retrieved all abstracts from Cochrane Colloquia that were available before the year 2021. We analyzed 8297 conference abstracts accepted at 25 Cochrane Colloquia, organized in the period 1994–2020, which were publicly available on the website of the Cochrane Library, in the section titled “Abstracts of oral and poster presentations, and workshops (from most past Colloquia)” [6].

### Eligibility

We created a list of all analyzed Cochrane Colloquium conference abstracts and screened each abstract to determine whether the abstract was promissory, i.e. without any results reported. We also considered promissory abstracts that described studies based on literature searches (i.e. systematic reviews, methodological studies analyzing literature) that reported only search results and no results regarding the study objectives.

Abstracts reporting minimal results or results of preliminary/interim analyses were not considered promissory. We also did not consider as promissory abstracts reporting descriptive commentaries/perspectives without research study methods reported. If the abstracts were designated as oral presentations or workshops, we carefully screened them to analyze whether they presented the results of original studies.

We excluded abstracts for which text was completely missing (only title and authors were provided), and abstracts for which part of the text was missing. In addition, we provided web references of such cases for transparency. We also excluded duplicate abstracts.

### Screening

One author screened all abstracts, while the second author randomly screened 10% of all abstracts and, in addition, all abstracts that were categorized as promissory or unclear by the first author.

### Data extraction

We extracted the following data from promissory abstracts: conference city, conference year, title, type of presentation (i.e., oral, poster, etc.), number of authors, number of unique countries in affiliations, affiliation countries using the “whole count” method in which each country got one mention when it appears in the affiliation of an author even if it was used multiple times for multiple authors, and self-reported study design.

### Data analysis

We used descriptive statistics. We presented data as frequencies and percentages. The number of authors was expressed as the median and interquartile range (IQR). For data analysis, we used Microsoft Excel (Microsoft Corp., Redmond, WA, USA).

## Results

We analyzed 8297 abstracts submitted to 25 Cochrane Colloquia from 1994 to 2020. The list of Colloquia, and the number of abstracts presented at each is presented in Table 1. Two abstracts were written in the French language [7, 8], and the rest were in English.

**Table 1:** The list of Cochrane Colloquia from which abstracts were analyzed

Cochrane Colloquium	Abstracts, N	Promissory abstracts, N (%)
1994 Hamilton	46	4 (8.7)
1995 Oslo	61	7 (11.5)
1996 Adelaide	103	17 (16.5)
1997 Amsterdam	220 (194 without duplicates)	20 (10.3)^a^
1998 Baltimore	108	5 (4.6)
1999 Rome	171	18 (10.5)
2000 Cape Town	209	24 (11.5)
2002 Stavanger	79	6 (7.6)
2004 Ottawa	268	20 (7.5)
2005 Melbourne	316	36 (11.4)
2006 Dublin	261	20 (7.7)
2007 Sao Paulo	136	7 (5.1)
2008 Freiburg	332	8 (2.4)
2009 Singapore	185	9 (4.9)
2010 Keystone	304	27 (8.9)
2011 Madrid	465	37 (8)
2012 Auckland	308	22 (7.1)
2013 Quebec City	612 (593 without duplicates)	44 (7.4)^a^
2014 Hyderabad	350	28 (8)
2015 Vienna	651	46 (7.1)
2016 Seoul	441	24 (5.4)
2017 Cape Town [Global Evidence Summit]	954	95 (10)
2018 Edinburgh	594	61 (10.3)
2019 Santiago	538	42 (7.8)
2020 Abstracts	585	90 (15.4)

^a^Percentage calculated from the number of abstracts without duplicates

Among the 8297 abstracts available on the Cochrane Library, we excluded 89 abstracts because they were duplicates (*N* = 43) or not evaluable (*N* = 46). The abstracts were not evaluable either because the abstract text was completely missing (4 cases) or part of the abstract was missing, so the abstract was not evaluable (42 cases). The list of excluded abstracts, with reasons, is provided in Supplementary file 1.

**Fig. 1:**
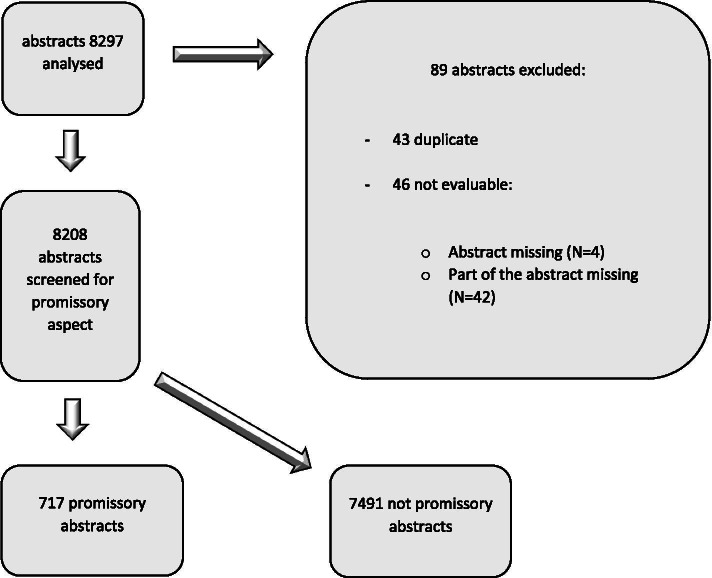
Study flow chart depicting the process of selecting promissory abstracts

The remaining 8208 abstracts were analyzed. A flow chart of abstracts’ inclusion is presented in Fig. 1.

**Fig. 2:**
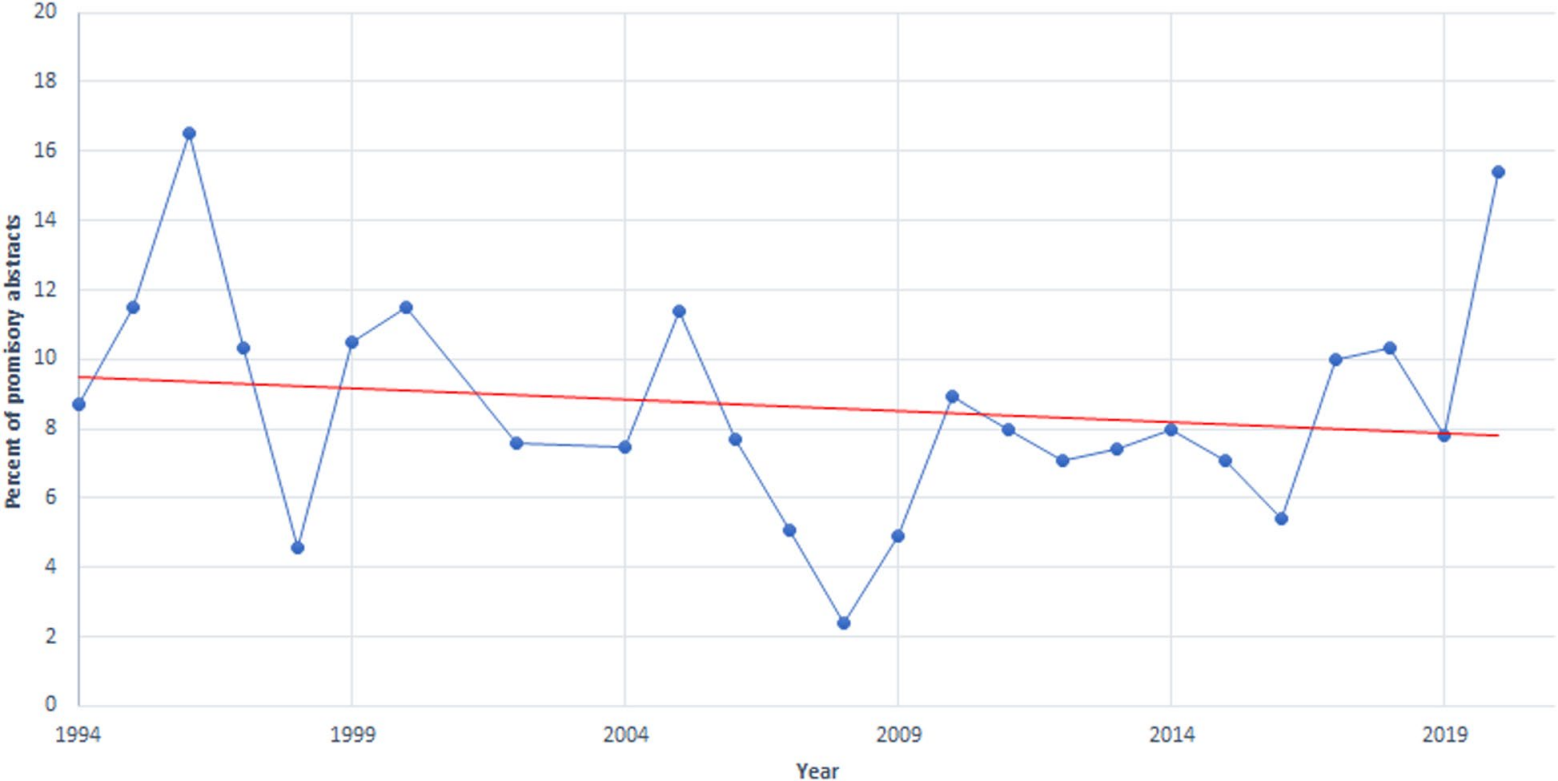
Percent of promissory abstracts in Cochrane Colloquia in 1994–2002 and the trendline (red line)

Among the analyzed abstracts, there were 717 (8.7%) promissory abstracts. The percentage of promissory abstracts among the abstracts presented at Colloquia over the years ranged from 2.1 to 16.5% (Table 1, Fig. 2). The trendline indicates that overall the number of promissory abstracts is decreasing over time (Fig. 2; red line).

Among 717 promissory abstracts, 475 (66%) were accepted as poster presentations, 241 (34%) as oral presentations, and 1 as a workshop. The abstract that described this future workshop indicated that results associated with the topic would be presented at the conference.

The median number of authors in promissory abstracts was 4 (IQR: 3 to 6 authors). The number of authors ranged from 1 to 26.

In 245 (34%) promissory abstracts, affiliations of authors were not reported. In the remaining 472 promissory abstracts, authors were affiliated with a total of 56 different countries. The authors were most commonly affiliated with the following countries: UK (169; 35.8%), Canada (*N* = 123; 26%), China (*N* = 76; 16.1%), United States (*N* = 66; 14%) and Australia (*N* = 53; 11.2%). A table with all affiliations is in Supplementary file 2.

There were 512 (71%) promissory abstracts in which study design was not reported. In the remaining 205 promissory abstracts, the authors used 75 different descriptors for their study designs. The most common self-reported study designs of promissory abstracts were systematic review (*N* = 58; 28.3%), scoping review (N = 12; 5.9%), cross-sectional study (*N* = 11; 5.4%), case study (*N* = 9; 4.4%), meta-analysis (N = 9; 4.4% and randomized controlled trial (*N* = 7; 3.4%). A table with all study designs is in Supplementary file 3.

Among the 58 studies self-described as systematic reviews, some were explicitly described as Cochrane reviews. An example is an abstract titled „A Cochrane Collaboration systematic review of melanoma incidence in randomized controlled trials of lipid-lowering agents“. While some of the systematic reviews were devoted to clinical questions, others addressed methodological aspects, for example „How to search practice guidelines efficiently: systematic review“. Details of these abstracts, together with all raw data collected within the study, are provided in Supplementary file 4.

## Discussion

Among the analyzed Cochrane Colloquia abstracts, 8.7% were promissory, i.e. without results. The trend indicates that the number of promissory abstracts is generally decreasing over the years in the analyzed 25 conferences. We could not find other reports in the literature analyzing the frequency and characteristics of promissory conference abstracts.

Results of many types of scientific studies are presented at professional meetings, and their summary is available in conference abstracts. The value of abstracts, both those from conferences and abstracts from scholarly publications, is well recognized. Conference abstracts are often the first report about a study, and journal abstracts may be the only information accessible to readers due to paywalls. There are multiple reporting guidelines for abstracts that can help authors of abstracts and help readers access transparent information about the studies. In 2008, CONSORT (Consolidated Standards of Reporting Trials) for Abstracts that report randomized controlled trials [9] was published. PRISMA (Preferred Reporting Items for Systematic Reviews and Meta-Analyses) for Abstracts, which was originally published in 2013 to foster transparent reporting of systematic review abstracts [10], was updated in 2020 [11]. It has been shown that uptake of these reporting guidelines may not have been optimal [12–14], but this will hopefully improve.

Multiple studies have shown that data reported in conference abstracts may not be reported in full-text scholarly articles many years after the conferences [15–17], and thus conference abstracts may be the only public record about a study being conducted. In the absence of full research reports, the abstracts may also be used in systematic reviews, as a source of information about eligible studies. Although conference abstracts are associated with particular issues, such as preferential publication of positive results, i.e. publication bias, systematic reviewers are urged to at least consider the availability of evidence informing the review [18].

However, the concept of promissory abstracts, i.e. abstracts only promising to deliver some results by the time the conference is organized, is not beneficial for researchers who are looking forward to reading results in the abstract books. The authors of the promissory abstract may indeed prepare results to be presented at a poster, oral talk or a workshop during the conference. However, none of the results will be included in the abstract book, as these books are prepared based on the abstracts that were submitted before the conference.

Our finding that promissory abstracts are very prevalent could prompt changes in the way abstract books are prepared. For example, organizers of all conferences that allow submission of promissory abstracts should request the authors to deliver the updated abstracts, with results, by the specific date. In this way, the abstract books would not include the “promissory” version of the abstract but the version with results instead. Some conferences indeed expect this from the authors of promissory abstracts. For example, The Society of Thoracic Surgeons (STS) provided the following instructions for authors submitting abstracts to the STS 2022 Annual Meeting: “*If you are involved in Phase I, II, or III clinical trials for which no preliminary data will be available by the August 3 deadline, you can submit a promissory abstract (data must be available by December 13, 2021)*.” [19].

Our study also points to some areas for improvement of Cochrane Colloquium abstracts. Firstly, we found several abstracts published in the French language. Both of these abstracts were accepted for a Cochrane Colloquium organized in Quebec City, Canada. With the full appreciation that Canada’s two official languages are English and French, publishing all Cochrane abstracts in the English language would be preferable for wider reach. Furthermore, for some Cochrane Colloquium abstracts, the text was completely missing, and for some abstracts part of the text was completely missing. A number of abstracts were duplicate, with different web links pointing to clearly duplicate abstracts. These findings can help curators of Cochrane Colloquium abstracts in cleaning their online files.

Future studies could analyze whether the authors indeed present their data from the promissory abstract at a conference, and whether there is any difference in the publication rate between the promissory and non-promissory conference abstracts. Also, it would be interesting to analyze the frequency and characteristics of promissory abstracts in non-Cochrane conferences. However, the problem with planning such studies is that conference abstracts are often not available to the public.

### Strengths

To our best knowledge, this is the first study on the frequency and characteristics of promissory abstracts at a series of major conferences. Our findings may help conference organizers to consider the potentially detrimental effect of promissory abstracts in the dissemination of knowledge from conferences. We also hope that our study may help Cochrane in improving the content and presentation of Cochrane Colloquia abstracts.

### Limitations

In this study, we relied on our subjective assessment when categorizing the abstracts as promissory or not. However, for transparency of our judgments, we have reported all our categorizations and verbatim extracts for the abstracts categorized as promissory in a supplementary file. Thus, readers can easily scrutinize our categorization.

Additionally, it would be worthwhile to analyze also submitted abstracts to see how many submitted promissory abstracts (if any) were rejected. However, this was not possible because information obtained from Cochrane in May 2020 indicated that the organization does not maintain a record of rejected abstracts from past events or details about reviewer scores.

Some of the abstracts in our sample could not be analyzed. In addition, many of the issues were specific for certain Cochrane Colloquia only. For example, cases of abstracts with partial text missing belonged to the years 2017 and 2018. Abstracts whose text was completely missing were from the years 1999, 2017 and 2020. Duplicate abstracts were observed only at conferences from 1997 and 2013. It could be concluded, thus, that these errors are not systemic but limited to certain conferences.

Of note, the present study only analyzed the abstracts presented at Cochrane Colloquia. Thus, the results may not be generalizable to abstracts from other conferences.

## Conclusion

Promissory abstracts were commonly accepted at the Cochrane Colloquia. Such abstracts deserve further attention, as their abstracts are not informative to the readers, and they are detrimental in terms of disseminating new knowledge presented at a conference. Conference organizers could ask the authors to update the abstract results subsequently to enable the dissemination of information presented at a conference.

## Supplementary Information

**Additional file 1:.** List of excluded abstracts, with reasons

**Additional file 2:.** Countries of author affiliations

**Additional file 3:.** Self-reported study designs in promissory abstracts

**Additional file 4:.** Raw data collected and analyzed within the study (available at Open Science Framework: https://osf.io/hwsa6/).

## Data Availability

Raw data generated in this study are available in the Supplementary file 4.
