# Supplementary material for: Frequency and characteristics of promissory conference abstracts, i.e. abstracts without results, accepted at Cochrane Colloquia 1994-2020
Source: BMC Med Res Methodol. 2021 Nov 8;21:243. doi: 10.1186/s12874-021-01442-3 (PMC8573995; doi:10.1186/s12874-021-01442-3)
Supplement: Supplementary file 1 — Additional file 1:. List of excluded abstracts, with reasons [file 12874_2021_1442_MOESM1_ESM.docx]

**Supplementary file 1. List of excluded abstracts, with reasons**

Hereby we provide a list of 89 excluded abstracts, which were excluded because they were duplicate (N=43) (**Table S1**) or not evaluable (N=46). The abstracts were not evaluable either because the abstract text was completely missing (4 cases) (**Table S2**) or part of the abstract was missing so the abstract was not evaluable (42 cases) (**Table S3**).

**Contents**

[**Table S1. The list of 43 duplicate abstracts** 1](#_Toc83889210)

[**Table S2. The list of 4 abstracts with text completely missing** 7](#_Toc83889211)

[**Table S3. 42 abstracts were part of the abstract was missing so the abstract was not evaluable (text cut as if only portion of the text was submitted)** 8](#_Toc83889212)

# **Table S1. The list of 43 duplicate abstracts**

| **N** | **Title and link** | **Cochrane Colloquium** |
| --- | --- | --- |
| 1 | "Admissible Evidence": the nature of evidence in Public Health and Medicine  <https://abstracts.cochrane.org/1997-amsterdam/admissible-evidence-nature-evidence-public-health-and-medicine-0> | 1997 Amsterdam |
| 2 | Evidence-based medicine: view from the trenches  <https://abstracts.cochrane.org/1997-amsterdam/evidence-based-medicine-view-trenches-0> | 1997 Amsterdam |
| 3 | The medical management of children who failed the Ewing hearing test: current practice compared to outcomes of RCTs  <https://abstracts.cochrane.org/1997-amsterdam/medical-management-children-who-failed-ewing-hearing-test-current-practice-compared-0> | 1997 Amsterdam |
| 4 | When clinical practice does not follow the evidence: physician's recommendation of mammography  <https://abstracts.cochrane.org/1997-amsterdam/when-clinical-practice-does-not-follow-evidence-physicians-recommendation-mammograp-0> | 1997 Amsterdam |
| 5 | Routine preoperative investigation: results of a survey in Italy  <https://abstracts.cochrane.org/1997-amsterdam/routine-preoperative-investigation-results-survey-italy-0> | 1997 Amsterdam |
| 6 | Current status and future perspectives of health technology assessment in Germany  <https://abstracts.cochrane.org/1997-amsterdam/current-status-and-future-perspectives-health-technology-assessment-germany-0> | 1997 Amsterdam |
| 7 | Promoting the rigorous development of clinical guidelines through the creation of a common appraisal  <https://abstracts.cochrane.org/1997-amsterdam/promoting-rigorous-development-clinical-guidelines-through-creation-common-appraisa-0> | 1997 Amsterdam |
| 8 | The UK NHS HTA programme  <https://abstracts.cochrane.org/1997-amsterdam/uk-nhs-hta-programme-0> | 1997 Amsterdam |
| 9 | Disseminating and implementing practice guidelines: findings from the U.S. Agency for Health Care Policy & Research  <https://abstracts.cochrane.org/1997-amsterdam/disseminating-and-implementing-practice-guidelines-findings-us-agency-health-care-0> | 1997 Amsterdam |
| 10 | The state of the art of practice guidelines development, dissemination, and evalution in Canada  <https://abstracts.cochrane.org/1997-amsterdam/state-art-practice-guidelines-development-dissemination-and-evalution-canada-0> | 1997 Amsterdam |
| 11 | Disseminating research findings to professional and lay audiences: a case study  <https://abstracts.cochrane.org/1997-amsterdam/disseminating-research-findings-professional-and-lay-audiences-case-study-0> | 1997 Amsterdam |
| 12 | Implementation of evidence-based indications for prevention of cardiovascular disease at a community level: an integrated project in the metropolitan area of Naples  <https://abstracts.cochrane.org/1997-amsterdam/implementation-evidence-based-indications-prevention-cardiovascular-disease-communi-0> | 1997 Amsterdam |
| 13 | A rough guide to implementation: the Royal College of General Practitioners (RCGP) in Scotland  <https://abstracts.cochrane.org/1997-amsterdam/rough-guide-implementation-royal-college-general-practitioners-rcgp-scotland-0> | 1997 Amsterdam |
| 14 | Demonstrating evidence-based practice  <https://abstracts.cochrane.org/1997-amsterdam/demonstrating-evidence-based-practice-0> | 1997 Amsterdam |
| 15 | Effectiveness of cholesterol testing and treatment in primary prevention  <https://abstracts.cochrane.org/1997-amsterdam/effectiveness-cholesterol-testing-and-treatment-primary-prevention-0> | 1997 Amsterdam |
| 16 | Developing cost effectiveness in guidelines  <https://abstracts.cochrane.org/1997-amsterdam/developing-cost-effectiveness-guidelines-0> | 1997 Amsterdam |
| 17 | Management strategies for peptic ulcer disease and Helicobacter pylori eradication  <https://abstracts.cochrane.org/1997-amsterdam/management-strategies-peptic-ulcer-disease-and-helicobacter-pylori-eradication-0> | 1997 Amsterdam |
| 18 | Economic evaluation of lung transplantation: results and implementation  <https://abstracts.cochrane.org/1997-amsterdam/economic-evaluation-lung-transplantation-results-and-implementation-0> | 1997 Amsterdam |
| 19 | Increasingly effective: a new resource to support effective communication with consumers  <https://abstracts.cochrane.org/1997-amsterdam/increasingly-effective-new-resource-support-effective-communication-consumers-0> | 1997 Amsterdam |
| 20 | Evidence-based patient choice: patients' and experts' views of patient information materials  <https://abstracts.cochrane.org/1997-amsterdam/evidence-based-patient-choice-patients-and-experts-views-patient-information-0> | 1997 Amsterdam |
| 21 | A population based study of patient treatment preferences for prostate cancer: early results from the Prostate Cancer Intervention Versus Observation Trial (PIVOT) Registry  <https://abstracts.cochrane.org/1997-amsterdam/population-based-study-patient-treatment-preferences-prostate-cancer-early-results-0> | 1997 Amsterdam |
| 22 | Prioritisation of varicose vein treatments - incorporating patient preferences  <https://abstracts.cochrane.org/1997-amsterdam/prioritisation-varicose-vein-treatments-incorporating-patient-preferences-0> | 1997 Amsterdam |
| 23 | Outcomes for assessing interventions to promote patient involvement in health care decisions  <https://abstracts.cochrane.org/1997-amsterdam/outcomes-assessing-interventions-promote-patient-involvement-health-care-decisions-0> | 1997 Amsterdam |
| 24 | Breast cancer screening: effective and efficient as well as efficacious  <https://abstracts.cochrane.org/1997-amsterdam/breast-cancer-screening-effective-and-efficient-well-efficacious-0> | 1997 Amsterdam |
| 25 | Superfilters website: a searching tool for review authors  <https://abstracts.cochrane.org/2013-qu%C3%A9bec-city/superfilters-website-searching-tool-review-authors-0> | 2013 Québec City |
| 26 | Epistemonikos: a comprehensive, systematic, collaborative and multilingual database for evidence-based health care  <https://abstracts.cochrane.org/2013-qu%C3%A9bec-city/epistemonikos-comprehensive-systematic-collaborative-and-multilingual-database-0> | 2013 Québec City |
| 27 | Shortening the pipeline: the use of data mining to link new trials to Cochrane Reviews  <https://abstracts.cochrane.org/2013-qu%C3%A9bec-city/shortening-pipeline-use-data-mining-link-new-trials-cochrane-reviews-0> | 2013 Québec City |
| 28 | Surveillance system assessing the need for updating systematic reviews  <https://abstracts.cochrane.org/2013-qu%C3%A9bec-city/surveillance-system-assessing-need-updating-systematic-reviews-0> | 2013 Québec City |
| 29 | Searching a database of knowledge translation resources for public health: The Registry of Methods and Tools  <https://abstracts.cochrane.org/2013-qu%C3%A9bec-city/searching-database-knowledge-translation-resources-public-health-registry-methods-0> | 2013 Québec City |
| 30 | DynaMed summaries and Cochrane Reviews: a dynamic collaboration!  <https://abstracts.cochrane.org/2013-qu%C3%A9bec-city/dynamed-summaries-and-cochrane-reviews-dynamic-collaboration-0> | 2013 Québec City |
| 31 | Making Cochrane Reviews more clinically accessible: the new Cochrane clinical answers derivative product  <https://abstracts.cochrane.org/2013-qu%C3%A9bec-city/making-cochrane-reviews-more-clinically-accessible-new-cochrane-clinical-answers-0> | 2013 Québec City |
| 32 | Development and evaluation of a point-of-care tool for providers based on a meta-analysis and clinical practice guideline  <https://abstracts.cochrane.org/2013-qu%C3%A9bec-city/development-and-evaluation-point-care-tool-providers-based-meta-analysis-and-0> | 2013 Québec City |
| 33 | The Healthcare Knowledge Integrity Framework: a conceptual map of the synergy, mediators, and threats to integrity within the healthcare research/practice continuum  <https://abstracts.cochrane.org/2013-qu%C3%A9bec-city/healthcare-knowledge-integrity-framework-conceptual-map-synergy-mediators-and-0> | 2013 Québec City |
| 34 | Systematic Review Data Repository (SRDR): beyond old school data abstraction  <https://abstracts.cochrane.org/2013-qu%C3%A9bec-city/systematic-review-data-repository-srdr-beyond-old-school-data-abstraction-0> | 2013 Québec City |
| 35 | Enhancing the efficiency of the systematic review process for evidence-based medicine  <https://abstracts.cochrane.org/2013-qu%C3%A9bec-city/enhancing-efficiency-systematic-review-process-evidence-based-medicine-0> | 2013 Québec City |
| 36 | Many hands make light work—or do they? Results of two pilot studies looking at the effects of crowdsourcing  <https://abstracts.cochrane.org/2013-qu%C3%A9bec-city/many-hands-make-light-work%E2%80%94or-do-they-results-two-pilot-studies-looking-effects-0> | 2013 Québec City |
| 37 | A cloud computing database for data extraction in a Cochrane Review  <https://abstracts.cochrane.org/2013-qu%C3%A9bec-city/cloud-computing-database-data-extraction-cochrane-review-0> | 2013 Québec City |
| 38 | Methods for configurational synthesis: extensions to traditional meta-analysis for addressing intervention complexity and contextual variation in reviews  <https://abstracts.cochrane.org/2013-qu%C3%A9bec-city/methods-configurational-synthesis-extensions-traditional-meta-analysis-addressing-0> | 2013 Québec City |
| 39 | Key domains of context and implementation and their assessment in systematic reviews of complex health interventions  <https://abstracts.cochrane.org/2013-qu%C3%A9bec-city/key-domains-context-and-implementation-and-their-assessment-systematic-reviews-0> | 2013 Québec City |
| 40 | Systematic reviews on multimorbidity: methodological challenges  <https://abstracts.cochrane.org/2013-qu%C3%A9bec-city/systematic-reviews-multimorbidity-methodological-challenges-0> | 2013 Québec City |
| 41 | Incorporation of assessments of risk of bias of primary studies in systematic reviews of randomized trials: a cross-sectional review  <https://abstracts.cochrane.org/2013-qu%C3%A9bec-city/incorporation-assessments-risk-bias-primary-studies-systematic-reviews-randomized-0> | 2013 Québec City |
| 42 | Reporting, dealing with, and judging risk of bias associated with missing participant data in systematic reviews: a methodological survey  <https://abstracts.cochrane.org/2013-qu%C3%A9bec-city/reporting-dealing-and-judging-risk-bias-associated-missing-participant-data-0> | 2013 Québec City |
| 43 | Do prominent biomedical journals have methods for detecting outcome reporting bias? (survey of the top 30 journals by impact factor)  <https://abstracts.cochrane.org/2013-qu%C3%A9bec-city/do-prominent-biomedical-journals-have-methods-detecting-outcome-reporting-bias-0> | 2013 Québec City |

# **Table S2. The list of 4 abstracts with text completely missing**

| **N** | **Title and link** | **Cochrane Colloquium** |
| --- | --- | --- |
| 1 | Priority setting for reviews and economic analyses at the World Health Organisation  <https://abstracts.cochrane.org/1999-rome/priority-setting-reviews-and-economic-analyses-world-health-organisation> | 1999 Rome |
| 2 | Bad examples of Interference In the conduct and publication of clinical research  <https://abstracts.cochrane.org/1999-rome/bad-examples-interference-conduct-and-publication-clinical-research> | 1999 Rome |
| 3 | The use of mhealth to promote women’s empowerment and gender equity in a rural health system in Burkina Faso  <https://abstracts.cochrane.org/2017-global-evidence-summit/use-mhealth-promote-women%E2%80%99s-empowerment-and-gender-equity-rural-health> | [2017 Cape Town [Global Evidence Summit]](https://abstracts.cochrane.org/search/site?f%5B0%5D=field_year%3A28) |
| 4 | Outcomes-Based Education (OBE) Model for Nursing Colleges: An ASEAN Integration Initiative of Philippine Schools of Nursing  <https://abstracts.cochrane.org/2017-global-evidence-summit/outcomes-based-education-obe-model-nursing-colleges-asean-integration> | [2017 Cape Town [Global Evidence Summit]](https://abstracts.cochrane.org/search/site?f%5B0%5D=field_year%3A28) |

# **Table S3. 42 abstracts were part of the abstract was missing so the abstract was not evaluable (text cut as if only portion of the text was submitted)**

| **N** | **Title and link** | **Cochrane Colloquium** |
| --- | --- | --- |
| 1 | Postpartum period: Mothers’ knowledge and hesitancy regarding vaccinations  <https://abstracts.cochrane.org/2017-global-evidence-summit/postpartum-period-mothers%E2%80%99-knowledge-and-hesitancy-regarding> | [2017 Cape Town [Global Evidence Summit]](https://abstracts.cochrane.org/search/site?f%5B0%5D=field_year%3A28) |
| 2 | Guidelines and postpartum mothers’ behaviour concerning antibiotic therapy and bacterial resistance  <https://abstracts.cochrane.org/2017-global-evidence-summit/guidelines-and-postpartum-mothers%E2%80%99-behaviour-concerning-antibiotic> | [2017 Cape Town [Global Evidence Summit]](https://abstracts.cochrane.org/search/site?f%5B0%5D=field_year%3A28) |
| 3 | Measuring the patient safety culture in a pharmacy setting in China  <https://abstracts.cochrane.org/2017-global-evidence-summit/measuring-patient-safety-culture-pharmacy-setting-china> | [2017 Cape Town [Global Evidence Summit]](https://abstracts.cochrane.org/search/site?f%5B0%5D=field_year%3A28) |
| 4 | Univariable meta-regression may be more conservative compared to chi-square in sub-group analyses  <https://abstracts.cochrane.org/2017-global-evidence-summit/univariable-meta-regression-may-be-more-conservative-compared-chi-square> | [2017 Cape Town [Global Evidence Summit]](https://abstracts.cochrane.org/search/site?f%5B0%5D=field_year%3A28) |
| 5 | Improving the precision of search strategies for guideline surveillance  <https://abstracts.cochrane.org/2017-global-evidence-summit/improving-precision-search-strategies-guideline-surveillance> | [2017 Cape Town [Global Evidence Summit]](https://abstracts.cochrane.org/search/site?f%5B0%5D=field_year%3A28) |
| 6 | Bivariate network meta-analysis of diagnostic test accuracy studies synthesising multiple tests and multiple thresholds  <https://abstracts.cochrane.org/2017-global-evidence-summit/bivariate-network-meta-analysis-diagnostic-test-accuracy-studies> | [2017 Cape Town [Global Evidence Summit]](https://abstracts.cochrane.org/search/site?f%5B0%5D=field_year%3A28) |
| 7 | Two-year follow up in a breast-screening decision aid RCT: Retention of overdetection knowledge and other decision-making effects  <https://abstracts.cochrane.org/2017-global-evidence-summit/two-year-follow-breast-screening-decision-aid-rct-retention> | [2017 Cape Town [Global Evidence Summit]](https://abstracts.cochrane.org/search/site?f%5B0%5D=field_year%3A28) |
| 8 | Are there differences in results between Bayesian and Frequentist network meta-analyses  <https://abstracts.cochrane.org/2017-global-evidence-summit/are-there-differences-results-between-bayesian%C2%A0and%C2%A0frequentist%C2%A0network> | [2017 Cape Town [Global Evidence Summit]](https://abstracts.cochrane.org/search/site?f%5B0%5D=field_year%3A28) |
| 9 | Testicular cancer - methodological quality of clinical guidelines and systematic reviews  <https://abstracts.cochrane.org/2017-global-evidence-summit/testicular-cancer-methodological-quality-clinical-guidelines-and> | [2017 Cape Town [Global Evidence Summit]](https://abstracts.cochrane.org/search/site?f%5B0%5D=field_year%3A28) |
| 10 | Guideline use behaviours and needs of primary-care practitioners in China: A cross-sectional survey  <https://abstracts.cochrane.org/2017-global-evidence-summit/guideline-use-behaviours-and-needs-primary-care-practitioners-china> | [2017 Cape Town [Global Evidence Summit]](https://abstracts.cochrane.org/search/site?f%5B0%5D=field_year%3A28) |
| 11 | Randomised-controlled trials are particularly scarce and underpowered in the setting of rare diseases: Further acquisition of knowledge is needed  <https://abstracts.cochrane.org/2017-global-evidence-summit/randomised-controlled-trials-are-particularly-scarce-and-underpowered> | [2017 Cape Town [Global Evidence Summit]](https://abstracts.cochrane.org/search/site?f%5B0%5D=field_year%3A28) |
| 12 | Knowledge brokering: An organisational strategy to support evidence-informed public health  <https://abstracts.cochrane.org/2017-global-evidence-summit/knowledge-brokering-organisational-strategy-support-evidence-informed> | [2017 Cape Town [Global Evidence Summit]](https://abstracts.cochrane.org/search/site?f%5B0%5D=field_year%3A28) |
| 13 | Evidence-based practice for nebulised gentamicin for bronchiectasis  <https://abstracts.cochrane.org/2017-global-evidence-summit/evidence-based-practice-nebulised-gentamicin-bronchiectasis> | [2017 Cape Town [Global Evidence Summit]](https://abstracts.cochrane.org/search/site?f%5B0%5D=field_year%3A28) |
| 14 | Exploration of factors influencing the development of evidence-based practice in a medical centre in northern Taiwan  <https://abstracts.cochrane.org/2017-global-evidence-summit/exploration-factors-influencing-development-evidence-based-practice> | [2017 Cape Town [Global Evidence Summit]](https://abstracts.cochrane.org/search/site?f%5B0%5D=field_year%3A28) |
| 15 | Measuring the impact of a laboratory practice guideline on immunohistochemical assay validation: Results from multiple modalities  <https://abstracts.cochrane.org/2017-global-evidence-summit/measuring-impact-laboratory-practice-guideline-immunohistochemical-assay> | [2017 Cape Town [Global Evidence Summit]](https://abstracts.cochrane.org/search/site?f%5B0%5D=field_year%3A28) |
| 16 | The influence of funding source on study characteristics in the Australian New Zealand Clinical Trials Registry (ANZCTR)  <https://abstracts.cochrane.org/2017-global-evidence-summit/influence-funding-source-study-characteristics-australian-new-zealand> | [2017 Cape Town [Global Evidence Summit]](https://abstracts.cochrane.org/search/site?f%5B0%5D=field_year%3A28) |
| 17 | Effect of video demonstration on choice of epidural labour analgesia in a Nigerian tertiary hospital  <https://abstracts.cochrane.org/2017-global-evidence-summit/effect-video-demonstration-choice-epidural-labour-analgesia-nigerian> | [2017 Cape Town [Global Evidence Summit]](https://abstracts.cochrane.org/search/site?f%5B0%5D=field_year%3A28) |
| 18 | Does trial registration reduce research bias? A comparison of registered and unregistered trials in diabetes quality improvement interventions  <https://abstracts.cochrane.org/2017-global-evidence-summit/does-trial-registration-reduce-research-bias-comparison-registered-and> | [2017 Cape Town [Global Evidence Summit]](https://abstracts.cochrane.org/search/site?f%5B0%5D=field_year%3A28) |
| 19 | Effect of ondansetron in children with acute diarrhoeal illness and vomiting with some dehydration – a RCT in Kenyatta National Hospital  <https://abstracts.cochrane.org/2017-global-evidence-summit/effect-ondansetron-children-acute-diarrhoeal-illness-and-vomiting-some> | [2017 Cape Town [Global Evidence Summit]](https://abstracts.cochrane.org/search/site?f%5B0%5D=field_year%3A28) |
| 20 | Methodological tasks of clinical practice guidelines developed in Japan  <https://abstracts.cochrane.org/2017-global-evidence-summit/methodological-tasks-clinical-practice-guidelines-developed-japan> | [2017 Cape Town [Global Evidence Summit]](https://abstracts.cochrane.org/search/site?f%5B0%5D=field_year%3A28) |
| 21 | Course of serological outcomes in treated subjects with chronic Trypanosoma cruzi infection: A meta-analysis of individual participant data conducted in Argentina  <https://abstracts.cochrane.org/2017-global-evidence-summit/course-serological-outcomes-treated-subjects-chronic-trypanosoma-cruzi> | [2017 Cape Town [Global Evidence Summit]](https://abstracts.cochrane.org/search/site?f%5B0%5D=field_year%3A28) |
| 22 | AHRQ EPC Methods Report: Characterising research evidence needs of hospitals and healthcare systems in the US  <https://abstracts.cochrane.org/2017-global-evidence-summit/ahrq-epc-methods-report-characterising-research-evidence-needs-hospitals> | [2017 Cape Town [Global Evidence Summit]](https://abstracts.cochrane.org/search/site?f%5B0%5D=field_year%3A28) |
| 23 | Quality of Brazilian Ministry of Health clinical practice guidelines: How can we improve?  <https://abstracts.cochrane.org/2017-global-evidence-summit/quality-brazilian-ministry-health-clinical-practice-guidelines-how-can> | [2017 Cape Town [Global Evidence Summit]](https://abstracts.cochrane.org/search/site?f%5B0%5D=field_year%3A28) |
| 24 | Multiple strategy peer-taught evidence-based medicine course in a poor resource setting.  <https://abstracts.cochrane.org/2017-global-evidence-summit/multiple-strategy-peer-taught-evidence-based-medicine-course-poor> | [2017 Cape Town [Global Evidence Summit]](https://abstracts.cochrane.org/search/site?f%5B0%5D=field_year%3A28) |
| 25 | Poor reporting and variability in comparator interventions in behavioural trials: A systematic review of smoking cessation interventions  <https://abstracts.cochrane.org/2018-edinburgh/poor-reporting-and-variability-comparator-interventions-behavioural-trials-systematic> | 2018 Edinburgh |
| 26 | A scale for measuring evidence-searching capability: a development and validation study  <https://abstracts.cochrane.org/2018-edinburgh/scale-measuring-evidence-searching-capability-development-and-validation-study> | 2018 Edinburgh |
| 27 | The development of IN SUM: a database of systematic reviews on effects of child mental health and welfare interventions  <https://abstracts.cochrane.org/2018-edinburgh/development-sum-database-systematic-reviews-effects-child-mental-health-and-welfare> | 2018 Edinburgh |
| 28 | Completeness of reporting of randomised controlled trials including people with transient ischaemic attack (TIA) or stroke: a systematic review  <https://abstracts.cochrane.org/2018-edinburgh/completeness-reporting-randomised-controlled-trials-including-people-transient> | 2018 Edinburgh |
| 29 | Evaluation of ‘spin’ in systematic reviews of diagnostic accuracy studies in high impact factor journals  <https://abstracts.cochrane.org/2020-abstracts/evaluation-%E2%80%98spin%E2%80%99-systematic-reviews-diagnostic-accuracy-studies-high-impact-factor> | 2020 Abstracts |
| 30 | Reporting Bias in Imaging Diagnostic Accuracy Research: Update on Recent Evidence  <https://abstracts.cochrane.org/2020-abstracts/reporting-bias-imaging-diagnostic-accuracy-research-update-recent-evidence> | 2020 Abstracts |
| 31 | Dissemination of Cochrane Public Health evidence with targeted stakeholder messages using an email marketing service  <https://abstracts.cochrane.org/2020-abstracts/dissemination-cochrane-public-health-evidence-targeted-stakeholder-messages-using> | 2020 Abstracts |
| 32 | Development and pilot of a framework using automation and crowd-sourcing to identify and classify randomized controlled trials for rheumatoid arthritis drug therapy  <https://abstracts.cochrane.org/2020-abstracts/development-and-pilot-framework-using-automation-and-crowd-sourcing-identify-and> | 2020 Abstracts |
| 33 | Design, conduct, and analysis of safety among drug-related systematic reviews: A cross-sectional survey  <https://abstracts.cochrane.org/2020-abstracts/design-conduct-and-analysis-safety-among-drug-related-systematic-reviews-cross> | 2020 Abstracts |
| 34 | Will you fund my idea? A retrospective look at public nominations to the AHRQ EPC Program.  <https://abstracts.cochrane.org/2020-abstracts/will-you-fund-my-idea-retrospective-look-public-nominations-ahrq-epc-program> | 2020 Abstracts |
| 35 | Effectiveness of Psychotherapy for Geriatric Negative Emotions: A Systematic Review and Meta-analysis of 12 randomized controlled trials  <https://abstracts.cochrane.org/2020-abstracts/effectiveness-psychotherapy-geriatric-negative-emotions-systematic-review-and-meta> | 2020 Abstracts |
| 36 | Certainty of evidence and level of recommendation for complex versus simple interventions in Chilean clinical guidelines.  <https://abstracts.cochrane.org/2020-abstracts/certainty-evidence-and-level-recommendation-complex-versus-simple-interventions> | 2020 Abstracts |
| 37 | Updating systematic reviews can improve the precision of outcomes: a comparative study  <https://abstracts.cochrane.org/2020-abstracts/updating-systematic-reviews-can-improve-precision-outcomes-comparative-study> | 2020 Abstracts |
| 38 | Impact of reporting quality on risk of bias assessment in occupational health and safety trials  <https://abstracts.cochrane.org/2020-abstracts/impact-reporting-quality-risk-bias-assessment-occupational-health-and-safety-trials> | 2020 Abstracts |
| 39 | The effects of interventions preventing depression in children and adolescents: an overview of systematic reviews  <https://abstracts.cochrane.org/2020-abstracts/effects-interventions-preventing-depression-children-and-adolescents-overview> | 2020 Abstracts |
| 40 | The effects of interventions for ADHD in children and adolescents: An updated review of systematic reviews  <https://abstracts.cochrane.org/2020-abstracts/effects-interventions-adhd-children-and-adolescents-updated-review-systematic-reviews> | 2020 Abstracts |
| 41 | The Reporting and Methodological Quality of Meta-Analysis related to Interventions Published in the Leading Surgery Journals: Overview and Evidence Mapping  <https://abstracts.cochrane.org/2020-abstracts/reporting-and-methodological-quality-meta-analysis-related-interventions-published> | 2020 Abstracts |
| 42 | Empirical evaluation of five statistical models in meta-analyses of treatments of chronic medical conditions: a meta-epidemiological study  <https://abstracts.cochrane.org/2020-abstracts/empirical-evaluation-five-statistical-models-meta-analyses-treatments-chronic-medical> | 2020 Abstracts |
