# Supplementary material for: Frequency and characteristics of promissory conference abstracts, i.e. abstracts without results, accepted at Cochrane Colloquia 1994-2020
Source: BMC Med Res Methodol. 2021 Nov 8;21:243. doi: 10.1186/s12874-021-01442-3 (PMC8573995; doi:10.1186/s12874-021-01442-3)
Supplement: Supplementary file 2 — Additional file 2:. Countries of author affiliations [file 12874_2021_1442_MOESM2_ESM.docx]

**Supplementary file 2. Countries of author affiliations**

The table shows countries of authors’ affiliations reported in the 472 promissory abstracts

| **Country** | **N (%)** |
| --- | --- |
| UK | 169 (35.8) |
| Canada | 123 (26.0) |
| China | 76 (16.1) |
| United States | 66 (14.0) |
| Australia | 53 (11.2) |
| Switzerland | 44 (9.3) |
| Germany | 38 (8.1) |
| Norway | 28 (5.9) |
| Italy | 23 (4.9) |
| Netherlands | 22 (4.7) |
| Spain | 21 (4.4) |
| Denmark | 19 (4.0) |
| Austria | 19 (4.0) |
| Chile | 18 (3.8) |
| Argentina | 17 (3.6) |
| Lebanon | 14 (3.0) |
| South Africa | 12 (2.5) |
| Belgium | 11 (2.3) |
| Croatia | 10 (2.1) |
| Brazil | 10 (2.1) |
| France | 10 (2.1) |
| Sweden | 9 (1.9) |
| Colombia | 6 (1.3) |
| Portugal | 5 (1.1) |
| India | 5 (1.1) |
| Romania | 4 (0.8) |
| Japan | 4 (0.8) |
| Finland | 4 (0.8) |
| Mexico | 4 (0.8) |
| Costa Rica | 4 (0.8) |
| Malaysia | 3 (0.6) |
| Ireland | 3 (0.6) |
| Cameroon | 3 (0.6) |
| Nigeria | 3 (0.6) |
| Bahrain | 2 (0.4) |
| Saudi Arabia | 2 (0.4) |
| Mozambique | 2 (0.4) |
| Indonesia | 2 (0.4) |
| Singapore | 2 (0.4) |
| Nepal | 2 (0.4) |
| Israel | 2 (0.4) |
| Thailand | 2 (0.4) |
| Malta | 1 (0.2) |
| Greece | 1 (0.2) |
| Kenya | 1 (0.2) |
| Salvador | 1 (0.2) |
| South Korea | 1 (0.2) |
| New Zealand | 1 (0.2) |
| Ecuador | 1 (0.2) |
| Ghana | 1 (0.2) |
| Slovakia | 1 (0.2) |
| Poland | 1 (0.2) |
| Bangladesh | 1 (0.2) |
| Zimbabwe | 1 (0.2) |
| Palestine | 1 (0.2) |
| Peru | 1 (0.2) |
