# Supplementary material for: Frequency and characteristics of promissory conference abstracts, i.e. abstracts without results, accepted at Cochrane Colloquia 1994-2020
Source: BMC Med Res Methodol. 2021 Nov 8;21:243. doi: 10.1186/s12874-021-01442-3 (PMC8573995; doi:10.1186/s12874-021-01442-3)
Supplement: Supplementary file 3 — Additional file 3:. Self-reported study designs in promissory abstracts [file 12874_2021_1442_MOESM3_ESM.docx]

**Supplementary file 3. Self-reported study designs in promissory abstracts**

The table shows self-reported study designs reported in the 205 promissory abstracts

| **Study design** | **N (%)** |
| --- | --- |
| Systematic review | 58 (28.3) |
| Scoping review | 12 (6) |
| Cross-sectional study | 11 (5.9) |
| Case study | 9 (4.4) |
| Meta-analysis | 9 (4.4) |
| Randomized controlled trial | 7 (3.4) |
| Systematic review and meta-analysis | 5 (2.4) |
| Meta-epidemiological study | 4 (2.0) |
| Overview of systematic review | 4 (2.0) |
| Qualitative study | 3 (1.5) |
| Re-analysis of meta-analysis | 3 (1.5) |
| Qualitative systematic review | 3 (1.5) |
| Retrospective cohort study | 2 (1.0) |
| Randomized trial | 2 (1.0) |
| User-testing study | 2 (1.0) |
| Bivariate meta-analysis | 2 (1.0) |
| Systematic survey | 2 (1.0) |
| Review of systematic reviews | 2 (1.0) |
| Retrospective study | 2 (1.0) |
| Cumulative meta-analysis | 2 (1.0) |
| Cluster randomized trial | 2 (1.0) |
| Cluster randomized controlled trial | 2 (1.0) |
| Crowd-based randomized methods study | 2 (1.0) |
| Methodological survey | 2 (1.0) |
| Randomized study | 2 (1.0) |
| Prospective study | 2 (1.0) |
| Methodological study | 1 (0.5) |
| Systematic survey of randomized controlled trials | 1 (0.5) |
| Systematic review and case report | 1 (0.5) |
| Operational research study | 1 (0.5) |
| Retrospective cross-sectional comparative study | 1 (0.5) |
| Overview of reviews | 1 (0.5) |
| Systematic methodological survey | 1 (0.5) |
| Consumer priority survey | 1 (0.5) |
| Systematic review of randomized clinical trials | 1 (0.5) |
| Overview of the randomized trials | 1 (0.5) |
| Registry study | 1 (0.5) |
| Participatory realist review | 1 (0.5) |
| Review of qualitative studies | 1 (0.5) |
| Patient-oriented rapid review | 1 (0.5) |
| Systematic evaluation study | 1 (0.5) |
| Pilot study | 1 (0.5) |
| Systematic realist review | 1 (0.5) |
| Progress report | 1 (0.5) |
| Systematic review and network meta-analysis | 1 (0.5) |
| Prospective collaborative meta-analysis of trials | 1 (0.5) |
| Systematic scoping review | 1 (0.5) |
| Delphi study | 1 (0.5) |
| Re-analysis of meta-epidemiological studies | 1 (0.5) |
| At-home study | 1 (0.5) |
| Cross-sectional evaluation study | 1 (0.5) |
| Qualitative review | 1 (0.5) |
| Methodological case study | 1 (0.5) |
| Dose–response meta-analysis of prospective cohort studies | 1 (0.5) |
| Methodological review | 1 (0.5) |
| Empirical study | 1 (0.5) |
| Systematic analysis | 1 (0.5) |
| Qualitative systematic survey | 1 (0.5) |
| Systematic literature scoping review | 1 (0.5) |
| Randomized controlled clinical trial | 1 (0.5) |
| Systematic overview of the literature | 1 (0.5) |
| Environmental scan | 1 (0.5) |
| Cochrane methodology review | 1 (0.5) |
| Experience-based co-design study | 1 (0.5) |
| Methodological systematic review | 1 (0.5) |
| International stakeholder survey | 1 (0.5) |
| Systematic review of meta-epidemiological studies | 1 (0.5) |
| Rapid literature review | 1 (0.5) |
| Systematic review protocol | 1 (0.5) |
| Rapid review | 1 (0.5) |
| Multinational study | 1 (0.5) |
| Realist review | 1 (0.5) |
| Comparative meta-epidemiological study | 1 (0.5) |
| Critical interpretive synthesis | 1 (0.5) |
| Qualitative analysis | 1 (0.5) |
